# Impact of the Marie Curie Cancer Care Delivering Choice Programme in Somerset and North Somerset on place of death and hospital usage: a retrospective cohort study

**DOI:** 10.1136/bmjspcare-2013-000645

**Published:** 2014-05-16

**Authors:** Sarah Purdy, Gemma Lasseter, Thomas Griffin, Lesley Wye

**Affiliations:** 1 Centre for Academic Primary Care, School of Social and Community Medicine, University of Bristol, Bristol, UK; 2 School for Policy Studies, University of Bristol, Bristol, UK

**Keywords:** palliative care, hospitalization, cohort studies

## Abstract

**Objectives:**

The Marie Curie Cancer Care Delivering Choice Programme (DCP) aims to help palliative patients be cared for in their place of choice. In this study, new palliative care services delivered in two counties in England included end-of-life care coordination centres, an out-of-hours telephone line and discharge in-reach nurses. The study aimed to investigate the impact of DCP on place of death and hospital usage (emergency department (ED) and admissions).

**Methods:**

Retrospective cohort of all eligible palliative patients who died over a 6-month period in two counties (n=3594). Participants were those who died of conditions considered to be eligible for end-of-life care, as defined by the Public Health England National End of Life Care Intelligence Network. The sample included people who did and did not access DCP services. DCP service, hospital admission and ED use data, demographic and death data were collected on all eligible participants. Data were analysed using descriptive statistics and logistic regression.

**Results:**

After adjusting for potential confounders, those using Delivering Choice were at least 30% less likely to die in hospital or have an emergency hospital admission or ED visit in the last 30 or 7 days of life than those who did not.

**Conclusions:**

Recipients of DCP services were less likely to die in or use hospital services. Those considering new ways of providing end-of-life care could explore the possibility of adopting similar services and evaluating the outcomes from patient, carer and system perspectives.

## Introduction

Since the publication of the End of Life Care Strategy in the UK in 2008, there has been increasing emphasis on supporting people to die in their own homes.^1^ From 2008 to 2011, Marie Curie Cancer Care worked with local professionals to develop end-of-life services in several parts of the UK, including North Somerset (North Somerset) and Somerset, two counties in southwest England.^2^ All palliative patients, regardless of condition, were eligible for the Delivering Choice Programme (DCP), which included.

- An out-of-hours (OOH) advice and response line manned by specialist palliative care nurses from 17:00 to 1:00, weekends and bank holidays, who responded to calls from professionals, family carers and patients (Somerset).
- Two ‘front of house’ hospital-based end-of-life care discharge-in-reach nurses who identified patients who wanted a non-hospital death and facilitated fast discharges accordingly (Somerset).
- Two end-of-life care coordination centres (one in each county) that took referrals from community, hospital and hospice staff to organise packages of care including equipment, night nurses and personal carers. In both counties, personal care was supplied from local agencies, but North Somerset also had its own in-house generic support team.
- These services were supported by an electronic end-of-life care register (EOL register) to record advanced care wishes.

In addition, in North Somerset, two end-of-life care facilitators were employed. These staff provided specific training, primarily to care homes and community teams, on issues such as advance care planning, end-of-life care pathways, use of the electronic end-of-life care register and medication including syringe drivers. No patient-level outcome data were linked to this intervention.

The aim of this study was to investigate the impact of Delivering Choice on place of death and hospital admissions and emergency department (ED) use. A parallel qualitative evaluation has been published elsewhere.^3^

## Methods

The sample was a retrospective cohort of people who died between 1 September 2011 and 29 February 2012 in North Somerset and Somerset and whose deaths were expected and potentially eligible for end-of-life care according to criteria derived by the UK National End of Life Care Intelligence Network.^4^ Quantitative data on use of Delivering Choice services were collected and linked via NHS number, age and postcode by National Health Service analysts to the Primary Care Mortality Database, in-patient hospital data and ED data obtained from NHS Connecting for Health. All patient data were pseudonymised during linkage. Differences between Delivering Choice and non-Delivering Choice users for place of death, emergency hospital admissions and ED visits in the last 30 and 7 days of life were explored. In addition, length of time between date of first contact with a Delivering Choice service and date of death was calculated, as was a Charlson Comorbidity Index for those people where data on comorbidities were available.^5^ Univariable analyses and multivariable logistic regression were undertaken using STATA V.12. The regression models adjusted for a priori confounding factors, including gender, age, deprivation, cause of death and other DCP services received.

The study was approved by local NHS Research and Development managers as a service evaluation, therefore NHS Research Ethics Committee and Research Governance approvals were not required. However, the study was reviewed by the ethics committee of the Faculty of Medicine at the University of Bristol and relevant Caldicott permissions were obtained.

## Results

### Participant characteristics

From 1 September 2011 to 29 February 2012, 1022 people potentially eligible for end-of-life care died in North Somerset and 2572 in Somerset (table 1). The overall mean age at death was 81 (North Somerset) and 82 (Somerset) with comparable proportions of women (54% and 55%) and men (46% and 45%). Commonest causes of death were cancer (North Somerset 28%, Somerset 29%), heart disease (18%, 18%), respiratory disease (15%, 13%) and dementia (15%, 13%).

**Table 1:** Characteristics of end-of-life populations, place of death and hospital use

	North Somerset (n=1022) (%)*		Somerset (n=2572) (%)*	
Delivering choice	Yes	No	Yes	No
N	213	809	616	1956
Age at death (mean SD)	79.4 (10.7)	81.2 (12.8)	77.3 (12.5)	82.4 (11.4)
Gender male	109 (51)	361 (45)	301 (49)	864 (44)
Deprivation (index of multiple deprivation)				
Least deprived	67 (31)	242 (30)	94 (15)	259 (13)
Below average deprivation	63 (30)	229 (28)	213 (35)	631 (32)
Average deprivation	33 (15)	119 (15)	178 (29)	629 (32)
Above average deprivation	10 (5)	65 (8)	100 (16)	345 (18)
Most deprived	37 (17)	139 (17)	29 (5)	80 (4)
Not available	3 (1)	15 (2)	2 (0)	12 (1)
Charlson score†				
Number of individuals	168 (79)	506 (63)	513 (83)	1321 (68)
Weighted score	4.6	3.0	5.3	2.9
Cause of death				
Cancer	145 (68)	142 (18)	440 (71)	314 (16)
Heart disease	15 (7)	170 (21)	44 (7)	430 (22)
Cerebrovascular	9 (4)	78 (10)	19 (3)	218 (11)
Respiratory	14 (7)	142 (18)	32 (5)	308 (16)
Dementia	12 (6)	145 (18)	30 (5)	316 (16)
Other	18 (8)	132 (16)	51 (8)	370 (19)
Place of death				
Acute hospital	40 (19)	347 (43)	84 (14)	836 (43)
Home‡	88 (41)	225 (28)	337 (54)	779 (40)
Care home (not usual place of residence)	34 (16)	116 (14)	58 (9)	173 (9)
Hospice	34 (16)	30 (4)	98 (16)	55 (3)
Community hospital	N/A	N/A	31 (5)	31 (5)
Elsewhere	17 (8)	91 (11)	8 (1)	12 (1)
Hospital utilisation				
Patients with one or more emergency admissions <30 days (SD)	61 (29)	335 (41)	233 (38)	875 (45)
Mean emergency admissions per patient <30 days (SD) [median, min, max]	0.31 (0.52) [0,0,2]	0.47 (0.60) [0,0,3]	0.53 (0.69) [0,0,3]	0.45 (0.64) [0,0,7]
Patients with one or more emergency admission <7 days (SD)	13 (6)	179 (22)	60 (10)	467 (24)
Mean emergency admissions per patient <7 days (SD) [median, min, max]	0.07 (0.27) [0,0,2]	0.23 (0.43) [0,0,2]	0.11 (0.33) [0,0,2]	0.25 (0.46) [0,0,2]
Patients with one or more ED attendance <30 days (SD)	54 (25)	363 (36)	159 (26)	712 (36)
Mean ED attendance per patient <30 days (SD) [median, min, max]	0.27 (0.50) [0,0,4]	0.52 (0.64) [0,0,2]	0.39 (0.51) [0,0,3]	0.41 (0.60) [0,0,5]
Patients with one or more ED attendance <7 days	13 (6)	213 (26)	43 (7)	432 (22)
Mean ED attendance per patient <7 days (SD) [median, min, max]	0.07 (0.29) [0,0,2]	0.27 (0.47) [0,0,2]	0.07 (0.27) [0,0,2]	0.26 (0.43) [0,0,3]

*Figure in parentheses refers to percentage unless otherwise indicated.

†Charlson score was calculated for those patients who had a hospital admission.

‡Home includes a care home where this was recorded as usual place of residence.

ED, emergency department; N/A, not applicable.

### Using Delivering Choice

Of the 1022 individuals in North Somerset who were eligible, 213 (21%) used one or more Delivering Choice intervention, most frequently the care coordination centre (153, 15%), followed by entry on the EOL register (93, 9%) (table 2). Less than 5% of people had a key worker listed on the EOL register (34, 4%) or accessed a generic support worker (GSW) (25, 2%). Data on who used GSW services were only available for 101 of the potential 181 days of the study.

**Table 2:** Delivering choice service use

Service	North Somerset N=1022 (%)	Somerset N=2572 (%)
Care coordination centre	153 (15)	294 (11)
EOL register	93 (9)	331 (13)
Key worker on EOL register	34 (4)	156 (6)
Generic support worker*	25 (2)	N/A
Out of hours advice line	N/A	243 (9)
Discharge in reach nurse	N/A	144 (6)
Total people using DCP	213 (21)	616 (24)

*Data were only available for 101 days.

DCP, Delivering Choice Programme.

Of the 2572 people in Somerset who were eligible, 616 (24%) used one or more DCP intervention (table 2). The most frequently used were entry on the EOL register (331, 13%), the care coordination centre (294, 11%) and the OOH advice line (243, 9%). Fewer people had a key worker listed on the EOL register (156, 6%) or were seen by a discharge in-reach nurse (144, 6%).

A much higher proportion of DCP users died from cancer than non-DCP users (table 1). The distribution of deprivation scores relative to England as a whole was similar for DCP users and non-users in both counties. Data on comorbidities were only available for those who had a hospital admission during the 6 months before death. Among these people, those who accessed DCP services had higher levels of comorbidity than those who did not access DCP (table 1).

### Duration of use of services

In North Somerset, median time for accessing the care coordination centre was 8 days before death (IQR 4–21). In Somerset, median time for accessing the care coordination centre was 9.5 days before death (IQR 4–20), while median time to death after first contact for the discharge in-reach service was 6 days (IQR 2–23) and for the OOH line 10 days (IQR 2–31).

### Place of death

Those receiving Delivering Choice were less likely to die in hospital (table 1). In North Somerset, the OR for dying in hospital was 0.33 (95% CI 0.21 to 0.50, p≤0.001) after adjusting for gender, age, deprivation and cause of death (table 3). In Somerset, the OR was 0.20 (95% CI 0.17 to 0.27, p<0.001). Looking at individual interventions, in North Somerset those who used the care coordination centre were less likely to die in hospital (OR 0.42, 95% CI 0.25 to 0.69, p<0.001) as were those patients entered on the EOL register (OR 0.30, 95% CI 0.13 to 0.69, p=0.005). In Somerset, patients accessing the care coordination centre were less likely to die in hospital (OR 0.11, 95% CI 0.06 to 0.22, p<0.001) as were those who used the OOH advice line (OR 0.34, 95% CI 0.20 to 0.57, p<0.001) or were entered on the EOL register (OR 0.22, 95% CI 0.12 to 0.40, p<0.001).

**Table 3:** Impact of Delivering Choice services versus non-Delivering Choice services on deaths in hospital, emergency admissions and A&E use*

	North Somerset			Somerset		
	OR	95% CI	p Value	OR	95% CI	p Value
Death in hospital overall	0.33	0.21 to 0.50	<0.001	0.20	0.17 to 0.27	<0.001
Key worker	0.74	0.18 to 3.05	0.679	0.73	0.27 to 1.95	0.524
Care coordination centre	0.42	0.25 to 0.69	0.001	0.11	0.06 to 0.22	<0.001
Generic support workers	0.34	0.04 to 2.64	0.30	N/A	–	–
EOL register	0.30	0.13 to 0.69	0.005	0.22	0.12 to 0.40	<0.001
Out-of-hours advice line	N/A	–	–	0.34	0.20 to 0.57	<0.001
Discharge in reach nurses	N/A	–	–	1.6	0.98 to 2.60	0.06
Emergency admission <30 days	0.49	0.33 to 0.74	0.001	0.61	0.48 to 0.76	<0.001
Key worker	0.60	0.21 to 1.69	0.33	1.13	0.68 to 1.87	0.642
Care coordination centre	0.55	0.34 to 0.90	0.016	0.58	0.42 to 0.80	0.001
Generic support workers	0.35	0.08 to 1.59	0.175	N/A	–	–
EOL register	0.65	0.33 to 1.30	0.225	0.41	0.28 to 0.60	<0.001
Out-of-hours advice line	N/A	–	–	0.78	0.56 to 1.10	0.159
Discharge in reach nurses	N/A	–	–	4.15	2.68 to 6.43	<0.001
Emergency admission <7 days	0.22	0.12 to 0.44	<0.001	0.32	0.23 to 0.45	<0.001
Key worker	1.13	0.18 to 7.03	0.898	1.04	0.45 to 2.40	0.934
Care coordination centre	0.09	0.02 to 0.39	0.001	0.26	0.15 to 0.46	<0.001
Generic support workers	6.26	0.81 to 48.65	0.079	N/A	–	–
EOL register	0.39	0.11 to 1.34	0.136	0.57	0.33 to 0.98	0.043
Out-of-hours advice line	N/A	–	–	0.44	0.25 to 0.78	0.005
Discharge in reach nurses	N/A	–	–	1.54	0.95 to 2.50	0.081
A&E attendance <30 days	0.41	0.28 to 0.62	<0.001	0.66	0.51 to 0.85	0.001
Key worker	0.71	0.25 to 2.02	0.515	1.23	0.71 to 2.13	0.454
Care coordination centre	0.46	0.29 to 0.76	0.002	0.58	0.40 to 0.82	0.002
Generic support workers	0.45	0.10 to 2.01	0.295	N/A	–	–
EOL register	0.57	0.29 to 1.11	0.097	0.61	0.40 to 0.92	0.018
Out-of-hours advice line	N/A	–	–	0.60	0.41 to 0.87	0.007
Discharge in reach nurses	N/A	–	–	3.29	2.23 to 4.87	<0.001
A&E attendance <7 days	0.22	0.11 to 0.42	<0.001	0.32	0.22 to 0.47	<0.001
Key worker	1.16	0.17 to 7.84	0.878	0.85	0.32 to 2.20	0.730
Care coordination centre	0.15	0.05 to 0.43	<0.001	0.24	0.12 to 0.48	<0.001
Generic support workers	2.39	0.27 to 21.61	0.437	N/A	–	–
EOL register	0.38	0.12 to 1.27	0.116	0.79	0.44 to 1.41	0.427
Out-of-hours advice line	N/A	–	–	0.34	0.17 to 0.70	0.003
Discharge in reach nurses	N/A	–	–	1.25	0.74 to 2.11	0.427

*Adjusted for gender, age, deprivation and condition.

### Hospital admissions

In the last 30 days of life, emergency hospital admissions were proportionally lower in both counties among those receiving a Delivering Choice intervention compared with non-users (table 1). For both 30-day (North Somerset: OR 0.49, 95% CI 0.33 to 0.74, p<0.001; and Somerset: OR 0.61, 95% CI 0.48 to 0.76, p<0.001) and 7-day (North Somerset: OR, 0.22 95% CI 0.12 to 0.44, p<0.001; Somerset: OR 0.32, 95% CI 0.23 to 0.45, p<0.001) results, these findings persist after adjusting for gender, age, deprivation and cause of death (table 3). The two coordination centres appeared to be the most effective components of the interventions at both 30 days (North Somerset: OR 0.55, 95% CI 0.34 to 0.90, p=0.016; and Somerset: OR 0.58, 95% CI 0.42 to 0.80, p<0.001) and 7 days (North Somerset: OR 0.09, 95% CI 0.02 to 0.39, p<0.001; and Somerset: OR, 0.26 95% CI 0.15 to 0.46, p<0.001). The OOH advice line was associated with lower risk of admission in the last week of life only (OR 0.44, 95% CI 0.25 to 0.78, p=0.005). Entry on the EOL register in Somerset was associated with lower risk of admission (30 days: OR 0.41, 95% CI 0.28 to 0.60; 7 days: OR 0.57, 95% CI 0.33 to 0.98, p=0.043) but not in North Somerset. Readmissions after accessing the discharge in-reach service were low at 6%.

### ED visits

In both counties, attendance at ED in the last 30 days of life was proportionately lower among DCP users (table 1). For both 30-day (North Somerset: OR 0.41, 95% CI 0.28 to 0.62, p<0.001; and Somerset: OR 0.66, 95% CI 0.51 to 0.85, p<0.001) and 7-day (North Somerset: OR 0.22, 95% CI 0.11 to 0.42, p<0.001; and Somerset: OR 0.32, 95% CI 0.22 to 0.47, p<0.001) results, these findings persist after adjusting for confounders (table 3). Again, the Care Coordination Centres appeared to be the most effective component at 30 days (North Somerset: OR 0.46, 95% CI 0.29 to 0.76, p=0.002; and Somerset: OR 0.58, 95% CI 0.40 to 0.82, p=0.002) and 7 days (North Somerset: OR 0.15, 95% CI 0.05 to 0.43, p<0.001; and Somerset: OR 0.24, 95% CI 0.12 to 0.48, p<0.001). The OOH advice line was associated with lower risk of ED attendance in the last week only (OR 0.34, 95% CI 0.17 to 0.70, p=0.003).

## Discussion

Patients who used Delivering Choice services were less likely to die in hospital, have emergency admissions or visit ED within 30 or 7 days before death than those who did not use Delivering Choice. The two coordination centres appeared to have the biggest impact.

There are a number of limitations to this study. Randomisation of patients was not practical in this service development. We addressed potential confounders such as age, gender, deprivation and cause of death. However, there are other ways in which the patient groups may have differed. For example, we were unable to include data on comorbidities in the logistic regression analyses. It is also possible that patients referred to the Delivering Choice services were people that a district nurse or GP considered were suitable to die at home, or who possibly wished to die at home, and the service was used to help facilitate this. This potential confounding by indication would result in increased uptake of the service by those who might have died at home anyway. In view of the limitations of the study design, we have reported associations but can make no claims for causal relationships.

Less than a quarter of all potential patients accessed Delivering Choice services. Moreover, about two-thirds of Delivering Choice service users had cancer while only about 30% of the total eligible population died from this condition. It is possible that by including a high proportion of patients with cancer, the DCP achieved more home deaths. Indeed, in the years 2008–2010, which preceded the introduction of Delivering Choice, 26% of patients with cancer in North Somerset and 28% of patients with cancer in Somerset died at home compared with 18% and 20% of all deaths being at home.^6^ However, among all patients with DCP, the proportions of deaths at home were 41% and 54%. Even among patients with cancer this represented a substantial increase in proportions of people dying at home.

The most effective components of the DCPs in both counties appear to be the care coordination centres. A qualitative evaluation of the components of Delivering Choice identified a number of factors that contribute to further understanding of the effectiveness of the individual interventions.^2^ The care coordination centres in the two counties operated in different ways. The North Somerset centre had an in-house model that included a fast-track coordinator, nurse assessors and its own team of personal care workers (GSWs). This maximised their flexibility to respond to patient and family needs, and this was particularly valued by families.^3^ Co-location with social service staff meant that the centre was well placed to identify potential patients early in the referral process for end-of-life care. Although the Somerset Care Coordination Centre had the same key function of organising care packages as the North Somerset Care Coordination Centre, the model was different. The Somerset Care Coordination Centre was led by a nurse and staffed exclusively by administrators, without any in-house care staff, additional nurses or fast-track coordinator. Thus to make this model work, the Somerset centre was heavily reliant on high-quality management and good external relationships, particularly with community and palliative care nurses and private care agencies. However, both services were valued for providing fast and efficient access to care packages and equipment. Previous systematic reviews have provided conflicting evidence with suggestions that specialist palliative care input at home can reduce general healthcare use and increase family and patient satisfaction with care but not all the evidence is supportive of this.^7^ ^8^

As only a third of patients accessing Delivering Choice had non-cancer diagnoses, this would suggest that primary healthcare teams should be encouraged to identify more patients for a palliative care approach and service providers should also redesign services that are manifestly appropriate for non-malignant patients. The study also highlights the difficulties of indentifying palliative patients in a timely way as half of Delivering Choice patients came into contact with the services just 6–20 days before death. Previous research in Scotland has identified that encouraging GPs to identify palliative care patients can be beneficial as 60% of patients on the GP palliative care register died at home, whereas 60% of people who were not on the palliative care register died in hospital.^9^ ^10^ However, GPs find early identification of non-cancer palliative care patients to be particularly challenging.^11^ Therefore, other interventions to improve the identification of patients for palliative care need to be put in place, in addition to providing services such as Delivering Choice for those already identified to be at the end of life. If services can identify and care for patients earlier, there is a possibility that hospital use at the end of life could be reduced further in line with national policy.^1^

In future studies, further evaluation of the components of the DCP that seemed to be most beneficial would be useful.^2^ Although randomised controlled trials of complex interventions are costly and difficult to undertake, the addition of trial evidence to the current literature would provide more robust evidence for those who commission and deliver services.

## Conclusion

The results from this evaluation suggest that patients using or supported by Delivering Choice were less likely to die in hospital or call on hospital services. Those considering new ways of providing end-of-life care could explore the possibility of adopting similar services and evaluating the outcomes from patient, carer and system perspectives.
